# Knowledge, acceptance and utilisation of the female condom among women of reproductive age in Ghana

**DOI:** 10.1186/s40834-017-0042-9

**Published:** 2017-04-18

**Authors:** Mark Kwame Ananga, Nuworza Kugbey, Jemima Misornu Akporlu, Kwaku Oppong Asante

**Affiliations:** 1 Department of Population and Behavioural Sciences, School of Public Health, University of Health and Allied Sciences, Hohoe, Volta Region Ghana; 2 Department of Family and Community Health, School of Public Health, University of Health and Allied Sciences, Hohoe, Volta Region Ghana; 3 Adidome District Health Directorate, Ghana Health Service, Ho, Volta Region Ghana; 4 Department of Psychology, School of Social Sciences, University of Ghana, Accra, Ghana

**Keywords:** Female condom, Knowledge, Acceptance, Utilisation

## Abstract

**Background:**

The female condom (FC) is the only safe and effective female-initiated method that provides simultaneous protection against unintended pregnancy as well as sexually transmitted infections (STIs), including HIV/AIDS. Knowledge of FC use among women and the perceptions and attitudes towards condom use can contribute to its uptake as an important public health strategy for HIV prevention in Ghana. However, there is a dearth of empirical evidence in this area of public health research to inform interventions. This study seeks to examine women’s knowledge, acceptance and utilisation of the FC and factors that influence its acceptance and utilisation.

**Methods:**

A descriptive cross-sectional survey design was used and a total of 380 females between the ages of 15 and 49 years were sampled from the Hohoe Municipality of the Volta Region, Ghana. A self-administered structured questionnaire measuring the study variables was used, and frequencies, percentages and Chi Square tests were used to analyse the data.

**Results:**

There is low level of FC use among the women as less than half (48.4%) of the sample were aware of the FC. It was further observed that 21.1, 21.8 and 11.1% of the sample reported friends, media and a public lecture as their sources of knowledge of the FC respectively. It was also observed that there is a low level of FC acceptance and utilisation, and also limited access to the FC from nearby shops/pharmacies (1.8%) and health centres (7.4%).

**Conclusions:**

There is a generally low level of FC awareness, knowledge, acceptance and utilisation and therefore, there is the need for increased public education on the FC and its benefits to women in preventing unwanted pregnancies and sexually transmitted diseases (STDs).

## Background

The global effort to curb the spread of HIV and other sexually transmitted infections (STIs) has resulted in the introduction of female condoms to empower women to take charge of their sexual and reproductive health issues. This is because of the belief that the female condom (FC) offers women double protection against sexually transmitted diseases such as HIV and unwanted pregnancies [1–5]. The consequences of unwanted pregnancies include unsafe abortions which present another public health challenge to the community and the country at large. Thus, the uptake of the FC is seen as one of the safest methods to reduce the risk of unwanted pregnancies and infection prevention. The Ghana AIDS Commission estimated in 2012 that there is a 2.1% HIV prevalence amongst pregnant women attending Antenatal clinic [6]. However, a decline in HIV prevalence of 1.9 in 2013 was attributed to the collaborative efforts by the Ghana AIDS Commission, Ghana Health Service, Non-governmental bodies and other stakeholders who have intensified their efforts aimed at reducing HIV among women and the population at large [6] Some of these efforts include intensified education on female condom and its effectiveness in protecting women again unwanted pregnancies and sexually transmitted infections.

Several negative consequences have been found to be associated with lack of contraceptive use among women most especially the use of the FC and some of these include unwanted pregnancies which predispose these women to severe socioeconomic and psychological challenges [7–9]. In addition, these women could contract chronic sexually transmitted diseases (STDs) such as HIV/AIDS, Hepatitis, Chancroid, Trichomoniasis, Human Papillomavirus (HPV) and Genital Warts among others, which could have severe consequences for the health and wellbeing of these women and their unborn babies in cases of pregnancy. A recent study among women of reproductive age in Ghana on the trends of contraceptive usage/practices showed that condom use was the least reported contraceptive practice as most women preferred to use unobservable contraceptives such as Depot Medroxyprogesterone [10]. Interestingly, the same study did not mention FC use even though it is thought that the condoms offer the safest and most effective protection against STIs and unwanted pregnancies.

In the Ghanaian context, as in other African countries, there are difficulties associated with women requesting for and carrying condoms due to misconceptions about condoms [10, 11] and other cultural influences which create gender-based inequality in condom use, which underpin the spread of HIV and AIDS [12, 13]. This gendered inequality is emphasised by the results from a study among Ghanaian women which found that condom use among both rural and urban women is very low [14, 15]. In a recent study conducted in Ghana, women’s perceived benefits of condom use and speaking to partners about how to avoid HIV predicted condom usage [10]. The same study also emphasized low rate of condom use among Ghanaian women of reproductive age [10]. This trend of low condom use among females has significant implications for the health and wellbeing of the population of women and the nation as a whole.

Research conducted in the sub-Saharan region and other parts of the world have identified several factors associated with the acceptance and utilisation of the FC among women of reproductive age [16–19]. Evidence suggests that there is generally a low level of knowledge about the FC, as Chipfuwa et al. [18] found among Zimbabwean women of reproductive age, that knowledge of the FC was low (36.3%) and most respondents (83.5%) reported never using them. The same study further revealed that unavailability of the FC and partner refusals were the key determinants of use [18]. These results are supported by findings from a review of studies on FC knowledge, acceptance and usage that male partner objection was the most commonly cited factor preventing initial and continued use of the FC [5]. Relatedly, low usage of the FC was reported in a sample of South African women over 15 years of age despite high knowledge of the FC [19]. The same study further reveled that locality, province, age, education level, marital status and employment status of the women sampled were significantly associated with knowledge of the FC while the actual utilisation of the FC was only predicted by province and age group [19]. On the other hand, positive attitudes, network exposure and peer influences and norms were found to be significantly associated with FC use among a sample of heterosexual males and females in the US, although overall uptake was very low [20].

In Ghana, there have been calls and effort for the re-launching of the FC by the Ministry of Health and Ghana Health Service, the two main bodies responsible for the healthcare needs of the country, as records at the Ghana Health Service indicate low patronage and usage of the FC among women of reproductive age. However, little attention has been paid to the barriers to FC knowledge, acceptability and utilisation among the women. This study sought to fill this gap by exploring FC knowledge, acceptance and utilisation in a sample of women of reproductive age to inform intervention measures aimed at increasing the acceptability and usage of the FC taking into cognizance its safety and effectiveness.

## Methods

The study targeted the adult population of women residing in Hohoe Municipality of the Volta Region in Ghana. The population of Hohoe Municipality, according to the 2010 Population and Housing Census, is 167, 016 representing 7.9% of the total population of the Volta Region. It comprises of 52.1% females and 47.9% males. About 52.6% of the population is urban. There are about 20 major towns/settlements making up the municipality. The total fertility rate for the municipality is 3.3. The general fertility rate is 96.0 births per 1 000 women aged 15 to 49 years. The mmunicipality has a household population of 164, 324 with 43, 329 households. The average household size in the mmunicipality is 3.9 persons per household (GSS, 2014).

The sample consisted of 380 women of reproductive age (18–49 years) and were included in the study if they fell within the age range, voluntarily gave consent to participate in the study and have resided in any of the communities in the Hohoe Municipality for the past year. A cross-sectional survey design was used and the women who consented to participate in the study were interviewed by a trained research assistant individually in their homes.

A self-developed questionnaire was used to gather information from the respondents on the study variables. The questionnaire was divided into four sections (A–D). Section A of the questionnaire comprised of the demographic characteristics of the respondents and these included age, marital status, years of formal education, employment status, religion and number of children. Section B of the questionnaire consisted of the respondents’ knowledge of the FC. Some of the issues considered included whether they have accurate knowledge of the importance of the FC in preventing unwanted pregnancies and STIs, ever seeing a pack of female condoms, and whether the FC is difficult to use/insert. Section C comprised of the acceptance of the FC by the respondents and issues covered in this domain included interference of the FC in the sexual act and other discomforts that could influence their acceptance of the FC. The Section D of the questionnaire comprised of FC utility or habits associated with use. Some of the issues covered included the women ever using the FC, frequency of use, spousal or partner approval of FC use and beliefs about FC use. Some items were also added to assess the accessibility of the condoms to the females in the municipality.

Ethical approval for the study was obtained from the Ethical Review Committee of the Ghana Health Service. The researchers adhered strictly to all the ethical issues involved in conducting research with human participants such as informed consent which was obtained after the aims and objectives of the study were explained to the respondents; confidentiality and anonymity were also ensured by not putting names or attaching any identifiable codes to the questionnaires, and the rights of the participants to withdraw from the study were also emphasised.

Data analysis was done with the use of the Statistical Package for the Social Sciences (SPSS) version 23. Frequencies, percentages and Chi Square tests were used to summarise the data with the alpha level set at .05.

## Results

### Demographic profile of the participants and female condom awareness

Results in Table 1 showed that the overall percentage awareness of FC usage among the women of reproductive age sampled for this study was 48.4%. Descriptive analysis showed that females between the ages of 18 and 25 years comprised of 23.7% of the sample while 39.5, 33.2 and 3.7% of the total sample were between the ages of 26 and 35 years, 36 and 45 years and 46 and 55 years respectively. In terms of the females’ awareness of FC use, it was observed that increasing age was associated with less awareness of FC use as 21.8, 19.2, 7.4 and 0.0% of awareness of FC use were reported by females between the ages of 15 and 25 years, 26 and 35 years, 36 and 45 years and 46 and 49 years respectively. The majority of the sample were married (62.5%) while 23.6, 4.0 and 9.9% of the sample were single, widowed and separated/divorced respectively. Married participants reported the highest percentage of awareness of FC use (24.7%) followed by 19.6, 8.0 and 0.0% by females who were single, separated/divorced and widowed respectively.

**Table 1:** Background characteristics of the participants and the percentage of awareness of female condom (FC) usage

Characteristics		Frequency	Percentage (%)	% Aware of FC use
Age groups				
	18–25years	90	23.7	21.8
	26–35years	150	39.5	19.2
	36–45years	126	33.2	7.4
	46–49years	14	3.7	0.0
Marital status				
	Single	83	23.6	19.6
	Married	220	62.5	24.7
	Widowed	14	4.0	0.0
	Separated/divorced	35	9.9	8.0
Years of education				
	No formal education	154	40.5	11.1
	6 years of education	112	29.5	16.6
	>6 years of education	114	30.0	20.8
Employment status				
	Informal	314	82.6	32.9
	Formal	31	8.2	8.2
	Student	35	9.2	7.4
Religion				
	Christianity	191	50.3	30.0
	Islam	112	29.5	11.1
	African Traditional	49	12.9	5.5
	Others	28	7.4	1.8
Number of children				
	None	86	22.6	20.8
	1-5children	259	68.2	27.6
	>5children	35	9.2	0.0

A substantial number of the sample (40.5%) had no formal education while 29.5 and 30.0% had 6 years of formal education and more than 6 years of formal education. More years of formal education was associated with increased awareness of FC use with only 11.1% of participants with no formal education reporting awareness of FC use while 16.6 and 20.8% of females with 6 years of formal education and more than 6 years of formal education respectively reported awareness of FC use. The mmajority of the sample were informally employed (82.6%) while only 8.2% were formally employed with the remaining 9.2% being students. Awareness of FC use was highest among the informally employed sample (32.9%), followed by formally employed (8.2%), with students reporting the least percentage of awareness of FC use (7.4%).

About half of the sample were Christians (50.3%) while the remaining half comprised of Muslims (29.5%), African Traditionalists (12.9%) and those who belong to other religions (7.4%). The percentage of awareness of FC use was highest among Christian respondents (30.0%) followed by Muslims (11.1%), African Traditionalists (5.5%), with the least reported awareness among participants who belong to other religions (1.8%). Most of the females in the study had between 1 and 5 children (68.2%), while the remaining 31.8% either had no child (22.6%) or had more than five children (9.2%). The percentage of awareness of FC use was highest among females who had between 1 and 5 children (27.6%), followed by females with no children (20.8%), while females with more than 5 children reported 0% awareness of FC usage.

### Female condom knowledge among the participants

Results from Table 2 below showed that majority of the females (70.5%) know that using the FC during sexual intercourse can prevent HIV and other STDs as well as unwanted pregnancy. However, a little above half of the participants (53.9%) reported to have even seen a pack of the condoms, while the remaining 46.1% of the sample reported to have never seen a pack of the condoms. However, less than half of the sample (47.6%) reported that the FC fresh from the pack cannot transmit an infection when used during sexual intercourse, with almost half of the sample (49.7%) reporting no knowledge on whether the FC fresh from the pack can transmit an infection when used during sexual intercourse or not, with only 2.6% reporting an erroneous impression that the FC fresh from the pack can transmit an infection when used during sexual intercourse. It was further observed that more than half of the sample (55.3%) reported no knowledge of the difficulty in using/inserting the FC while 40.3% were of the view that it is difficult to insert the FC, with only 4.5% of the sample reporting that the FC is not difficult to use/insert. The percentages suggest that the level of FC knowledge among the sample of these females is relatively low.

**Table 2:** Knowledge of the female condom among women of reproductive age

Statements	*N*	(%)	*ρ*-value
Using the female condom during sex can prevent HIV and other STDs			<.001
Yes	268	70.5	
No	7	1.8	
Don’t Know	105	27.6	
Using the female condom can prevent pregnancy			<.001
Yes	268	70.5	
No	7	1.8	
Don’t Know	105	27.6	
Ever seen a pack of female condoms before?			.124
Yes	205	53.9	
No	175	46.1	
The female condom fresh from the pack can transmit an infection when used during sexual intercourse			<.001
Yes	10	2.6	
No	181	47.6	
Don’t Know	189	49.7	
The female condom is difficult to use/insert			<.001
Yes	153	40.3	
No	17	4.5	
Don’t Know	210	55.3	

In terms of source of knowledge about the FC, 21.1% reported their friends as the source, 21.8% of the sample gained knowledge from the media, while 11.1% of the sample reported knowledge of FC from a public lecture. However, the remaining 46.1% did not indicate any source of knowledge about the FC. It was also observed that only 9.2% of the total sample reported receiving advice/education from their doctor/nurse on FC.

### Acceptance of the female condom among women in their reproductive ages

Results in Table 3 below showed that 44.9% of the sample reported that the FC interferes with their sexual pleasure/sensation while only 1.8% of the sample disagreed that the FC interferes with their sexual pleasure/sensation. The remaining 54.2% of the sample did not know whether the FC interferes with their sexual pleasure/sensation. A substantial percentage of the sample (41.1%) reported using a FC during sex is not comfortable, while only 3.7% of the sample disagreed that using a FC during sex is not comfortable. The remaining 55.3% of the sample did not know whether using a FC during sex is not comfortable.

**Table 3:** Acceptance of female condom among women in their reproductive ages

Statements	*N*	(%)	*ρ*-value
Using the female condom interferes with my sexual pleasure			<.001
Agree	167	43.9	
Disagree	7	1.8	
Don’t know	206	54.2	
Using a female condom during sex is not comfortable			<.001
Agree	156	41.1	
Disagree	14	3.7	
Don’t know	210	55.3	
The female condom is too wet or too slippery			<.001
Agree	174	45.8	
Disagree	7	1.8	
Don’t know	199	52.4	
The female condom has an unpleasant scent			<.001
Agree	28	7.4	
Disagree	142	37.4	
Don’t know	210	55.3	
The female condom makes noise when used during sexual intercourse			.002
Agree	160	42.1	
Don’t know	220	57.9	

Further, 45.8% of the sample agreed that the FC is too wet or too slippery while only 1.8% disagreed. The remaining 52.4% of the sample did not know whether the FC is too wet or too slippery. Only 7.4% of the sample agreed that the FC has an unpleasant scent while 37.4% of the sample disagreed that the FC has an unpleasant scent. However, the remaining 55.3% of the sample did not know whether the FC has an unpleasant scent or not. Finally, 42.1% of the sample agreed that the FC makes noise when used during sexual intercourse with the remaining 57.9% of the sample reporting no knowledge of whether the FC makes noise when used during sexual intercourse or not.

### Female condom use among women of reproductive age

Results in Table 4 below show that 40.3% of the sample agreed that their spouse/main partners do not like them to use the FC during sex while only 4.5% of the sample disagreed that their spouse/main partners do not like them to use the FC during sex, which suggests low female condom practice/utilisation. An appreciable percentage of the sample (33.7%) agreed that if they have sex with other partner(s) they do not like them to use the FC with only 3.7% of the sample disagreeing that if they have sex with other partner(s) they do not like them to use the FC. These percentages also suggest low usage of the FC among the sample. However, 42.1% of the sample prefers it when their spouse/partners ask them to use the FC while only 6.3% of the sample indicated otherwise should their spouse/main partners ask them to use a FC, which suggests that the use of the FC can be improved with partner/spousal support.

**Table 4:** Female condom use among women of reproductive age

Statements	*N*	%	*ρ*-value
My spouse/main partner does not like me to use the female condom during sex			<.001
Agree	153	40.3	
Disagree	17	4.5	
Don’t know	210	55.2	
If I have sex with other partner(s) they do not like me to use the female condom			<.001
Agree	128	33.7	
Disagree	14	3.7	
Don’t know	238	62.6	
I do not like it if my spouse/main partner asks me to use a female condom			<.001
Agree	24	6.3	
Disagree	160	42.1	
Don’t know	196	51.6	
I insert/put on the female condom before I start any sexual act as a measure to prevent unwanted pregnancy			<.001
Never	217	57.1	
Sometimes	149	39.2	
Most of the times	14	3.7	
I insert/put on the female condom before I start any sexual act as a measure to prevent HIV and other STDs			<.001
Never	224	58.9	
Sometimes	142	37.4	
Most of the times	14	3.7	
Using a female condom means that I do not trust my partner			<.001
Agree	247	65.0	
Disagree	63	16.6	
Don’t know	70	18.4	

Interestingly, the majority of the sample (58.9%) never insert/put on the FC before they start any sexual act as a measure to prevent HIV and other STDs, while only 37.4 and 3.7% of the sample sometimes and always insert/put on the FC before they start any sexual act as a measure to prevent HIV and other STDs respectively. These percentages suggest low utilisation of the FC among the sample selected. It was also revealed that the majority of the sample (65.0%) agreed that using a FC means that “I do not trust my partner.” This trend has severe implications for women’s health in terms of contracting HIV and other STIs and unwanted pregnancies.

### Accessibility of the female condom

**Fig. 1:**
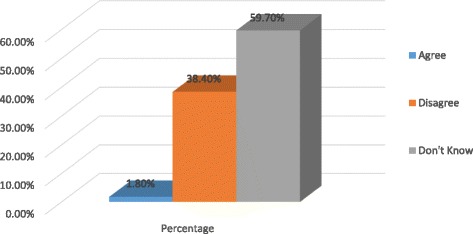
Percentages of accessibility of the female condom from a nearby shop/chemist

The study further examined the accessibility of the FC to the women and the results showed that only 1.8% of the total sample agreed that the FC is easily available from the nearby shop or chemist, while 38.4% of the sample disagreed that the FC is easily available from the nearby shop or chemist. However, the majority of the study participants did not know whether the female condom is easily available from the nearby shop or chemist as shown in the Fig. 1 below.

**Fig. 2:**
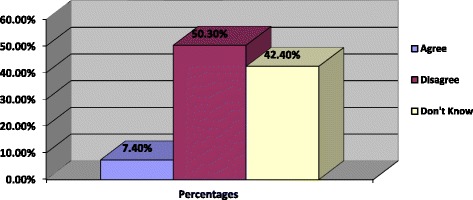
Percentages of accessibility of the female condom from a nearby health centre

It was also observed that only 7.4% of the sample agreed that the FC is easily available from the nearby health centre, with approximately half of the sample (50.3%) of the sample disagreeing that the FC is easily available from the nearby health centre. However, 42.4% of the sample reported that they did not know whether the FC is easily available from the nearby health centre or not. The summary of the results is presented in Fig. 2 below.

**Fig. 3:**
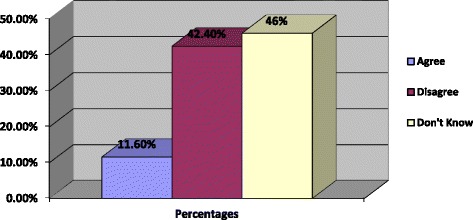
Percentages of whether the participants think the female condom is expensive

Further, the researchers also assessed whether the respondents thought that the FC is expensive and the results showed that 11.6% of the sample agreed that the FC is expensive, while 42.4% disagreed that the FC was expensive. However, 46% of the sample reported that they did not know whether the female condom is expensive or not. The summary of the results is presented in Fig. 3 below.

## Discussion

This study sought to examine FC knowledge, acceptance and utilisation among a sample of women of reproductive age (18–49 years) in the Volta Region of Ghana. This was necessitated by the fact that the FC offers women double protection against unwanted pregnancies and STDs. Findings from the study showed that less than half of the participants (48.4%) had overall awareness of FC usage among the women of reproductive age. This low percentage of reported awareness among women in the study implies that a substantial number of women are not aware of the FC and its relevance to their wellbeing despite the conscious attempts being made by the Ghana Health Service and the Ghana AIDS Commission to increase FC use among women in Ghana. This finding suggests that there is the need to create for further awareness which is targeted at regions outside the capital of Ghana. However, this finding is inconsistent with a previous finding among young women which showed a high level of FC awareness [21]. This inconsistency could be due to the sample compositions as this current study had a majority of participants who had no formal education or less than 6 years of formal education.

In this study, women’s knowledge of the FC and its uses were assessed and the findings showed that the level of knowledge among the sample of these women is relatively low. For instance, it was observed that 49.7% of the sample reported no knowledge on whether the FC fresh from the pack can transmit an infection when used during sexual intercourse or not while an additional 2.6% of the sample reported the erroneous impression that the FC fresh from the pack can transmit an infection when used during sexual intercourse. In the same vein, 46.1% of the sample reported that they have not seen a FC in a pack before. These examples suggest that FC knowledge among this sample of women is relatively low which calls for concerted efforts aimed at increasing FC awareness and knowledge among women in their reproductive ages. This finding of relatively low FC knowledge is consistent with the finding in a sample of Zimbabwean women of reproductive age that their knowledge of the FC is low [18]. This low knowledge could be attributable to the low level of awareness creation on the FC within the municipality.

Acceptance of the FC as a protective tool for unwanted pregnancies and STDs is likely to inform utilisation of the FC. With this background, the FC acceptance was examined among the sample and the findings show that 44.9% of the sample reported that the FC interferes with their sexual pleasure/sensation, 41.1% of the sample reported that using a FC during sex is not comfortable and 42.1% of the sample agreed that the FC makes noise when used during sexual intercourse. The noise from the female condom during sexual intercourse seems to be one of the key drawbacks for the acceptance of the product and therefore, requires consideration from the manufacturers and all stakeholders. These percentages of unfavorable attitudes could lead to low acceptance of the FC as the females in the study perceived the FC to be unpleasant. This requires thorough and vigorous public health action to increase awareness and acceptance of the FC as low acceptance of poses a danger to the health and wellbeing of the women and the nation as a whole. This relatively low level of FC acceptance was reported in a systematic review of FC acceptability in the sub-Saharan African region [22]. Similar findings were observed in Zimbabwe, where the acceptability of the FC is low due to its convenience in terms of ease of access and difficulty in inserting it [23].

The ultimate aim of every public health education or intervention is the utilisation of the information received by the recipients. Thus, the utilisation of the FC was assessed among this sample and the findings indicate that only 4.5% of the sample reported that their spouse/partners allow them to use the FC during sexual intercourse while only 3.7% were of the view that they are allowed by other sexual partners to use the FC during sexual intercourse. The non-acceptance of the female condom by males during sexual intercourse with their partners/spouses points to the unequal power balance between men and women within the Ghanaian and other African contexts where women are expected to be submissive to their male counterparts [24]. In addition, the low acceptance on the part of men could be due to the belief that their partners do not trust them and would want to avoid the condoms to prove their faithfulness. However, there is the need for educating men about the importance of female condom and also, a conscious effort to change their perceptions about female condom.

It was further observed that only 39.2% sometimes put on a FC, while only 3.7% of the sample put on a FC most of the time. These percentages suggest low FC utilisation which poses a serious public health challenge to the municipality and the country as a whole. This low utilisation of the condom is consistent with the finding in Ghana that the condom is one of the least employed contraceptive methods by women of reproductive age [10]. Similar findings of low FC utilisation have been reported by other researchers [4, 14, 19].

Evidence in Ghana suggests that condom knowledge has not yet transformed into its usage [15]. This lack of significant connection between condom knowledge and its utility has been attributed to cultural values, beliefs and practices that influence power relations in sexual practices and behaviours which are found in Sub-Saharan African countries and other minority cultures [12, 15, 24–28]. For instance, it has been established that intention to use condom among immigrants was largely influenced by their subjective cultural norms and beliefs [25]. These cultural practices include the perception of condom use as a sign of mistrust in one’s partner which could account for the limited utilization of the female condom among the women sampled in this study. The implication of this finding is that these women are likely to be at risk for STDs as well as unwanted pregnancies and their associated medical and psychosocial consequences.

The study also found that accessibility of the FC from nearby shops or pharmacies and health centres is extremely low among the sample as only 1.8% of the sample reported that the FC is accessible from nearby shops/pharmacies, while 7.4% reported the availability of the FC at health centers. However, the majority of the sample did not know whether the FC was available from nearby shops/pharmacies and health centres. It was further revealed that 11.6% of the sample reported that the FC is expensive while almost half of the sample did not know about the cost of the FC. This lack of access could affect acceptance of the FC and its usage as some earlier studies have reported [4, 18]. These findings imply that there is the need for increased access to the FC at pharmacies and health centres with a vigorous public education of the relevance of the FC to women in protecting them against unwanted pregnancies and STDs.

This study has some limitations that are worth a mention. Firstly, the cross-sectional and descriptive nature of the study makes for any causal inferences to be drawn between the study variables. In addition, the study was from only one municipality and therefore it cannot be generalized to the whole country. Despite these limitations, this study has provided an insight into what pertains in some parts of Ghana regarding FC knowledge, acceptance and utilisation which is likely to inform investigation in other municipalities as well. This will help in developing intervention programmes aimed at increasing FC awareness, acceptance and utilisation among women of reproductive age.

## Conclusion

The consequences of low knowledge, acceptance and utilisation of the FC among women of reproductive age are a major public health concern especially in a developing country like Ghana with its own socioeconomic difficulties. Findings from this study showed that FC knowledge, acceptance and utilisation among this sample of women is relatively low compared to studies elsewhere. This could partly be due to the very low accessibility of the FC from nearby pharmacies and health centres. The implication of these findings is that these women of reproductive age within the municipality require rigorous public education through the use of all available means to increase awareness and clear misconceptions about the FC. Conscious efforts should also be made at the health facilities to promote the FC to females of reproductive age to increase acceptance and usage in order to empower women in their sexual reproductive health.

## Abbreviations

AIDS: Acquired Immune Deficiency Syndrome
FC: Female condom
GSS: Ghana Statistical Service
HIV: Human immunodeficiency virus
STDs: Sexually transmitted diseases
STIs: Sexually transmitted infections
